# Sex Differences in the Development of Malignancies among End-Stage Renal Disease Patients: A Nationwide Population-Based Follow-Up Study in Taiwan

**DOI:** 10.1371/journal.pone.0044675

**Published:** 2012-09-05

**Authors:** Chi-Jung Chung, Chao-Yuan Huang, Hung-Bin Tsai, Chih-Hsin Muo, Mu-Chi Chung, Chao-Hsiang Chang, Chiu-Ching Huang

**Affiliations:** 1 Department of Medical Research, China Medical University and Hospital, Taichung, Taiwan; 2 Department of Health Risk Management, College of Public Health, China Medical University, Taichung, Taiwan; 3 Department of Urology, National Taiwan University Hospital, College of Medicine National Taiwan University, Taipei, Taiwan; 4 Department of Traumatology, National Taiwan University Hospital, Taipei, Taiwan; 5 Department of Public Health, China Medical University, Taichung, Taiwan; 6 Management Office for Health Data, China Medical University and Hospital, Taichung, Taiwan; 7 Division of Nephrology, Department of Internal Medicine, Taichung Veterans General Hospital, Taichung, Taiwan; 8 Department of Urology, China Medical University and Hospital, Taichung, Taiwan; 9 Department of Medicine, College of Medicine, China Medical University and Hospital, Taichung, Taiwan; 10 Division of Nephrology and Kidney Institute, China Medical University and Hospital, Taichung, Taiwan; University of Sao Paulo Medical School, Brazil

## Abstract

Increasing evidence indicates that end-stage renal disease (ESRD) is associated with the morbidity of cancer. However, whether different dialysis modality and sex effect modify the cancer risks in ESRD patients remains unclear. A total of 3,570 newly diagnosed ESRD patients and 14,280 controls matched for age, sex, index month, and index year were recruited from the National Health Insurance Research Database in Taiwan. The ESRD status was ascertained from the registry of catastrophic illness patients. The incidence of cancer was identified through cross-referencing with the National Cancer Registry System. The Cox proportional hazards model and the Kaplan–Meier method were used for analyses. A similar twofold increase in cancer risk was observed among ESRD patients undergoing hemodialysis (HD) or peritoneal dialysis (PD) after adjusting for other potential risk factors. Patients with the highest cancer risk, approximately fourfold increased risk, were those received renal transplants. Urothelial carcinoma (UC) had the highest incidence in HD and PD patients. However, renal cell carcinoma (RCC) had the highest incidence in the renal transplantation (RT) group. In addition, female patients undergoing RT or PD had a higher incidence of RCC and UC, respectively. Male patients under HD had both higher incidence of RCC and UC. In conclusion, different dialysis modality could modify the cancer risks in ESRD patients. We also found sex effect on genitourinary malignancy when they are under different dialysis modality.

## Introduction

End-stage renal disease (ESRD), as the fifth stage of chronic kidney disease (CKD), exemplifies progressive kidney damage. In Taiwan, chronic kidney disease was the tenth leading causes of death in 2010. From 1990 to 2007, the mortality rate increased from 11.4 to 22.2 per 100,000 persons (Department of Health, Executive Yuan, R.O.C.). Based on the United States Renal Data System (USRDS) Annual Data Report, Taiwan ranks first in the world in the incidence and prevalence of ESRD at 415 and 2288 per million population in 2007, respectively. Regular renal replacement therapy (RRT) with hemodialysis (HD), peritoneal dialysis (PD) or renal transplantation (RT) enhances the survival of patients with ESRD, but other complications might occur, including cardiovascular disease (CVD) [1]–[3]. These issues indicate that ESRD is a substantial burden to global health care and it reduces the quality of life of patients.

Until now, increasing evidence has indicated that CKD and ESRD are associated with the morbidity and mortality of cancer, including renal cell carcinoma (RCC), urothelial carcinoma (UC), and thyroid and lung cancer [4]–[6]. RCC and UC are the major malignancies among dialysis patients in Western countries and Taiwan, respectively [7], [8]. The choice of RRT or dialysis duration might modify the risk of cancer [9], [10]. Patients undergoing hemodialysis (HD) longer than ten years have greatly increased risk of developing RCC with a sarcomatoid component than those with shorter than ten years [11]. Moreover, the different histologic spectrum of RCC occurs according to the duration of dialysis [12]. Renal transplant recipients are known to have a higher cancer incidence compared with the general population [13], [14]. According to the USRDS, renal transplantation is related to the incidence of renal cell carcinoma[15]. However, the mechanism through which renal transplantation increases the incidence of cancer has not yet been fully described.

In one study on ESRD patients, the top three of the most common cancer sites among males and females were the liver, the colon, and the urinary tract, in descending order, followed by thyroid and lung [6]. In general, the progression of renal failure in patients with CKD is more rapid in men than in women [6], [16]. However, a recent study in Taiwan revealed that female ESRD patients have a higher risk of malignancy than male patients [17]. Furthermore, the gender effect on the relationship between dialysis modality and the renal outcome of patients with cancer remains unexamined.

The types of malignancies may occur differently because of distinct geographic regions and ethnic populations. Whether the comparison of dialysis modality or choice of transplantation also modifies the cancer outcomes in patients with ESRD remains unclear. Therefore, through a nationwide database, the effect of dialysis modality and renal transplantation on incident cancer morbidity was investigated and the interaction of gender effect and dialysis modality on survival among a cohort of patients with ESRD in Taiwan was assessed.

## Materials and Methods

The present study was constructed as a retrospective cohort study through an encrypted database from the Bureau of National Health Insurance (BNHI); therefore the Institutional Review Board (IRB) waved the need for informed consent for this study.

### Data source

The study used the Longitudinal Health Insurance Database (LHID) of the Taiwan National Health Research Institute (NHRI). The NHRI constructed insurant claims, which were released by the BNHI. The BNHI provided the medical claims data after scrambling the identification and this study was exempted by the IRB. The LHID included all medical records from 1996 to 2009, from which one million people were randomly selected from all insurant. There were no significant differences in the sex and age distributions between LHID and all insurant in the NHRI. The program was set up on March 1, 1995, with coverage of over 99.6% in 2009. The International Classification of Disease Revision 9th Clinical Modification (ICD-9-CM) was used for the diagnosis codes. Information about the databases has been detailed in a previous paper [18].

### Study sample

Total 4,032 patients that were initially diagnosed with ESRD (ICD-9-CM 585) were selected from the registry of catastrophic illness patients, and the index date was the date of ESRD registration. Patients with a history of malignant cancer (ICD-9-CM 140–208) before the index date (*N* = 246), with incomplete age or sex information (*N* = 34), or did not undergo dialysis (*N* = 253) were excluded. Finally, all 3,570 ESRD patients were included as the ESRD group. All subjects without a history of ESRD were randomly selected from all NHI beneficiaries. Four controls for each case were frequency matched for age 5 years each, sex, index month, and index year as the control group. All controls were screened using the same exclusion criteria used for the ESRD cases. We used the method of 1:*m* matching to screen the control subjects, which can increase the statistical power and control the potential confounding. Even if *m* > 4, the statistical efficiency did not considerably increase. Therefore, we constructed a 1∶4 matched study design. The ESRD group was divided into three subgroups according to RRT modality: the HD, PD and RT groups; those with a higher frequency of HD treatments were assigned to the HD group, and those with a higher frequency of PD treatments were assigned to the PD group. All ESRD subjects were followed-up until the date at which they were diagnosed with malignant cancer. The incidence of kidney transplantation (ICD-9-CM V420) was ascertained before the said date.

### Patient variables

Sociodemographic variables, such as age, sex, and level of urbanization, as well as comorbidities, such as hypertension (ICD-9-CM 401-405), diabetes (ICD-9-CM 250), and hyperlipidemia (ICD-9-CM 272) were analyzed. The level of urbanization of the patient residences was divided into two groups based on the NHRI report. All comorbidities were determined before the index date. Hypertension and hyperlipidemia were confirmed with at least three medical visits and diabetes by two visits within the first year of diagnosis.

### Statistical analysis

All statistical analyses were performed using the SAS statistical software (version 9.1 for Windows; SAS Institute, Inc., Cary, NC, USA). We described and compared the distributions of age, sex, Urbanization level (high and low), and co-morbidities (%) between controls and patients with various RRT modalities by χ^2^ tests. Person-year was calculated as the interval from the index date to the date of malignant cancer diagnosis, loss to follow-up, death, or the end of 2009. The incidence density (per 1,000 person-years) was assessed for each subgroup. Cox proportional hazard regression was used to estimate the hazard ratio (HR) of developing malignant cancer. The cumulative incidence of malignancy among the control group, HD group, and the PD group were assessed using the Kaplan–Meier method and the differences were estimated with a log-rank test.

## Results

### Baseline characteristics of the study subjects

The 3,570 ESRD patients consisted of 3,065 HD patients, 317 PD patients and 188 RT patients as well as 14,280 controls were recruited. Information on the demographic characteristics and co-morbidities of the ESRD patients by treatment status is provided in Table 1. The mean age of the ESRD patients was similar to the age of the normal control group. The PD group was younger than the HD group (*p*<0.0001). Gender distribution was well balanced in both controls and cases as well as in all study groups. Compared with the control group, the ESRD group has more percentage of hypertension, diabetes, and hyperlipidemia (*p*<0.0001).

**Table 1:** Demographic characteristics and co-morbidities of patients with end-stage renal disease by treatment status.

	End-stage renal disease											
Variables	ControlsN = 14,280		TotalN = 3,570		HDN = 3,065		PDN = 317			RTN = 188		p-value
	n	%	N	%	n	%	n	%	n		%	
Age, years												1.00
<40	1,164	8.2	291	8.2	172	5.6	62	19.6	57		30.3	
40–49	1,988	13.9	497	13.9	387	12.6	62	19.6	48		25.5	
50–59	3,096	21.7	774	21.7	638	20.8	81	25.6	55		29.3	
60–69	3,528	24.7	881	24.7	803	26.2	55	17.4	24		12.8	
≥70	4,504	31.5	1,126	31.5	1,065	34.8	57	18.0	4		2.1	
mean (SD)	60.9	(15.0)	61.3	(14.9)	63.0	(14.0)	53.5	(16.6)	46.4		(12.8)	0.13
Sex												1.00
Women	7,248	50.8	1,812	50.8	1,548	50.5	172	54.3	92		48.9	
Men	7,032	49.2	1,758	49.2	1,517	49.5	145	45.7	96		51.1	
Urbanization level												0.42
High	7,860	55.0	1,992	50.8	1,684	54.9	198	62.5	110		58.5	
Low	6,419	45.0	1,578	44.2	1,381	45.1	119	37.5	78		41.5	
Co-morbidities												
Hypertension	5,977	41.9	3,215	90.1	2,784	90.8	272	85.8	159		84.6	<0.0001
Diabetes	1,998	14.0	1,836	51.4	1,699	55.4	111	35.0	26		13.8	<0.0001
Hyperlipidima	3,357	23.5	1,699	47.6	1,511	49.3	132	41.6	56		29.8	<0.0001

p-values were evaluated through chi-square tests.

### Incidence and HR of all-site cancer according to treatment status

The mean duration of follow-up was 3.18 years in ESRD patients (3.13 years in HD patients, 2.96 years in PD patients and 4.3 years in RT patients) compared with 4.45 years in controls (Table 2). The incidence of malignancy among ESRD patients is higher by 1.74-fold than the control group. The highest incidence of malignancy was shown in RT patients. The adjusted hazard risk of malignancy was 1.92 among ESRD patients and the subgroups were 1.81, 2.07, and 3.83 in HD, PD, and RT patients, respectively (all *p*<0.05). Further stratification by follow-up duration revealed that the patients with the highest risk of malignancy among the different-duration groups from the shortest to the longest period were RT, RT, and PD patients, respectively.

**Table 2:** Incidence rate and hazard ratios of overall cancer according to treatment status for patients with end-stage renal disease.

	Controls	Total	HD	PD	RT
Mean duration of follow-up (yr)	4.45	3.18	3.13	2.96	4.30
Person-years at risk	63,583	11,341	9,595	934	808
Overall cancer					
No. of events	655	203	172	13	18
Incidence*	10.3	17.9	17.9	13.9	22.3
Model 1	1.00 (reference)	2.01 (1.71–2.36)	1.90 (1.61–2.25)	2.17 (1.25–3.77)	4.04 (2.51–6.50)
Model 2	1.00 (reference)	1.92 (1.61–2.30)	1.81 (1.50–2.19)	2.07 (1.19–3.62)	3.83 (2.36–6.20)
Follow-up duration					
<2.5	1.00 (reference)	1.52 (1.17–1.96)	1.51 (1.15–1.97)	1.09 (0.45–2.66)	2.71 (1.18–6.23)
2.5–5.4	1.00 (reference)	1.91 (1.40–2.61)	1.61 (1.14–2.26)	3.28 (1.33–8.08)	6.31 (3.26–12.2)
≥5.5	1.00 (reference)	3.53 (2.36–5.28)	3.52 (2.32–5.35)	5.46 (1.69–17.6)	2.31 (0.56–9.56)

* Per 1000 person-years.

Models were adjusted for the following variables: Model 1, age and sex; Model 2, age, sex, and medical history of hypertension, hyperlipidima, diabetes.

### Cause-specific cancer associated with ESRD patients receiving different RRT modalities

The HRs of cause-specific cancer associated with various ESRD treatments are shown in Table 3. Compared with the control group, the HR significantly increased among the ESRD and HD group patients for UC, followed by RCC, and thyroid cancer. In the PD group, the significant HRs were for UC. In the RT group, the highest risk was in RCC, followed UC.

**Table 3:** Hazard ratios and 95% confidence interval of cancer risks associated with end-stage renal disease in Cox's regression analysis in different cancers.

	Controls	Total		HD		PD		RT	
Cancer Type (ICD-9-CM)	Case	Case	HR (95% CI)	Case	HR (95% CI)	Case	HR (95% CI)	Case	HR (95% CI)
Overall cancer (140-208)	655	203	1.92 (1.61–2.30)	172	1.81 (1.50–2.19)	13	2.07 (1.19–3.62)	18	3.83 (2.36–6.20)
Oral cavity cancer (140–149)	32	5	1.03 (0.37–2.86)	4	0.99 (0.33–3.00)	0	–	1	2.27 (0.28–18.2)
Digestive									
All (150–159)	271	61	1.41 (1.04–1.92)	53	1.35 (0.98–1.87)	4	1.73 (0.64–4.69)	4	2.44 (0.89–6.67)
Colorectal cancer (153–154)	93	21	1.54 (0.92–2.59)	19	1.52 (0.89–2.60)	1	1.45 (0.20–10.6)	1	2.21 (0.30–16.3)
Liver cancer (155)	104	21	1.14 (0.67–1.92)	17	1.01 90.58–1.78)	2	2.04 (0.49–8.44)	2	2.64 (0.63–11.1)
Respiratory									
All (160–165)	126	14	0.75 (0.42–1.36)	11	0.65 (0.34–1.24)	2	2.02 (0.49–8.33)	1	1.59 (0.22–11.6)
Bone, skin and breast									
All (170–175)	70	16	1.24 (0.67–2.27)	13	1.16 (0.60–2.22)	1	0.99 (0.13–7.60)	2	2.86 (0.67–12.2)
Genitourinary									
All (179–189)	113	92	4.93 (3.56–6.84)	77	4.70 (3.35–6.60)	6	4.93 (2.12–11.5)	9	8.75 (4.24–18.0)
UC (188, 189.1–189.9)	35	53	13.3 (7.95–22.2)	44	12.5 (7.42–21.3)	5	19.0 (7.04–51.1)	4	17.9 (5.97–53.9)
RCC (189.0)	17	28	8.45 (4.16–17.2)	22	7.77 (3.73–16.2)	1	4.18 (0.53–32.9)	5	24.4 (7.87–75.6)
Other and unspecified									
All (190–199)	13	9	5.57 (2.02–15.3)	8	5.84 (2.06–16.5)	0	–	1	9.17 (1.06–79.4)
Thyroid cancer (193)	7	5	5.60 (1.42–22.1)	4	5.45 (1.28–23.2)	0	–	1	115.2 (1.51–153)
Haemopoietic									
All (200–208)	25	6	0.93 (0.35–2.44)	6	1.02 (0.39–2.70)	0	–	0	–

Models were adjusted for age, sex, and medical history of hypertension, hyperlipidima, and diabetes.

### The gender effect on the risk of all-site cancer, UC, and RCC

**Figure 1:**
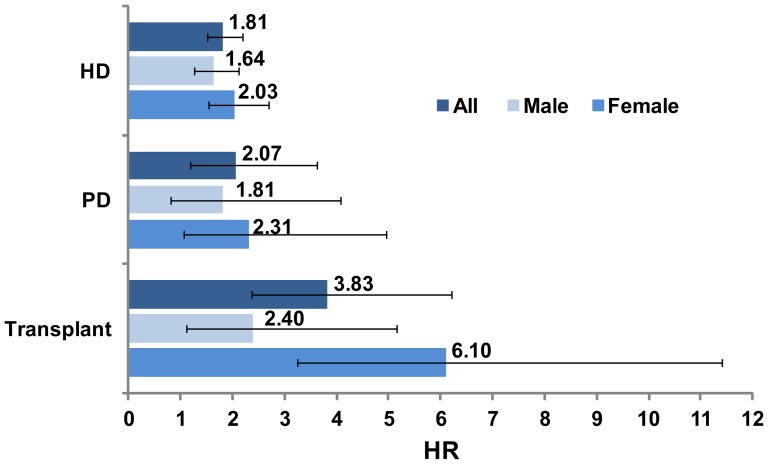
Hazard ratio and 95% confidence interval of overall cancer associated with end-stage renal disease in Cox's regression analysis by adjusted for age, sex, and medical history of hypertension, hyperlipidima, and diabetes.

**Figure 2:**
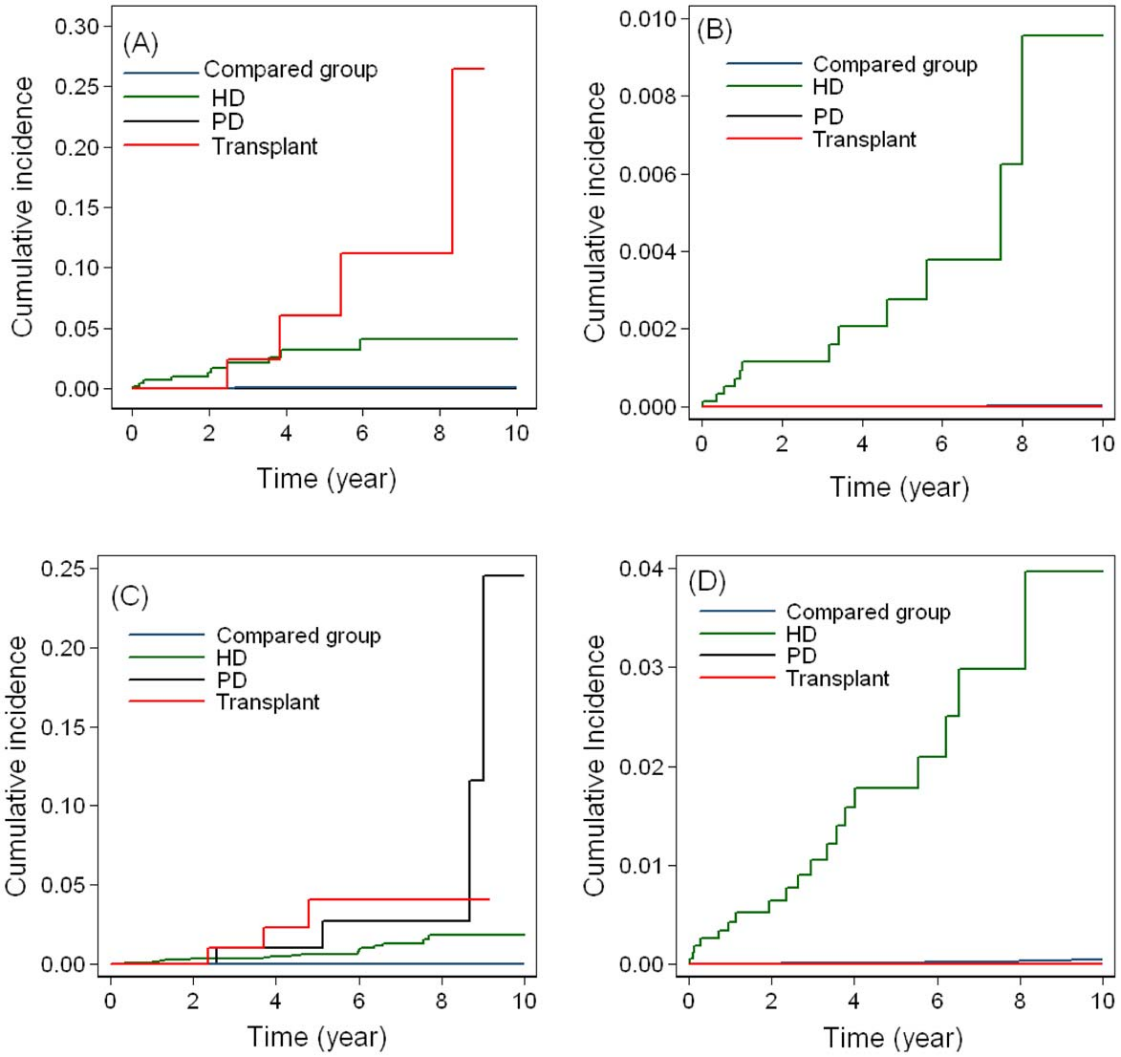
Cumulative incidence of cancer by sex after adjusting for age, hypertension, hyperlipidemia, and diabetes. (A) RCC in female, (B) RCC in male, (C) UC in female, (D) UC in male.

The highest incidence of overall cancers was noted in RT patients, followed by PD and HD patients after adjusted for potential risk factors (Figure 1). After stratification by sex, higher risk of cancer was noted in female patients in all RRT subgroups than in male patients. After considering the different RRT effects on the risks of UC or RCC, we found that the RT and PD groups had the highest incidence of RCC and UC among all the RRT subgroups, respectively, in the female group (data not shown). In the male group, those patients who underwent HD had both the relatively higher risk of kidney cancer and UC than the other subgroups (data not shown). After adjusting for age, hypertension, hyperlipidemia, and diabetes, similar results were obtained (Figure 2).

## Discussion

Thus far, the present study is the first to compare the effect of gender on susceptibility to malignancies in ESRD patients, as well as the effects of different RRT on cancer risks through national health database linkages in Taiwan. An approximately fourfold risk of cancer was observe among RT patients compared with the control group. Next, a similar twofold risk of all-site cancer was observed among ESRD patients undergoing HD or PD treatment. In addition, we also observed the gender effect in RRT-related malignancy. In female patients, those who have RT had higher risk of RCC and those having PD had higher risk of UC than those under other RRT. However, in male patients, those having HD had both higher risks of RCC and UC than those in other subgroups.

The cancer morbidity and mortality among patients with ESRD are reportedly higher compared with the general population even though they are receiving dialysis or renal transplantation [5], [9]. The exact pathogenesis is not yet fully understood. However, several reasons were proposed, including viral infection, a weakened immune system, chronic inflammation, immunosuppressive treatment, and impairment of DNA repair [19], [20]. An increased risk of morbidity of overall cancer is associated with longer dialysis duration in accordance with previous studies [5], [21]–[23]. However, above studies had not compared the effect of different RRT on cancer risk. It might be mostly because of a low percentage of PD treatments. Recently, a Korean study analyzed a cohort of 4,562 ESRD patients from hospital-based database, and indicated that among the 106 ESRD patients with cancer, 63 (59%) received HD and 43 received PD (41%) [6]. A recent study on another group in Taiwan indicated the significantly increase risk of post-transplantation malignancies using a standardized incidence ratio (SIR) based on the national health database [24]. The incidence rates of post-transplantation malignancies in our analysis were similar with the results of Li *et al* and the most common malignancies occurred in the urinary tract, which represent about 50% of the cancers diagnosed [24]. This suggests that urinary tract malignancies (RCC) are most common among the ESRD patients that received kidney transplants in the Taiwan area.

“Generally, male CKD or ESRD patients had worse progression of renal outcomes than women [6], [16], [25]. A total of 4,562 ESRD patients from Korea showed that male patients (73/2760) had more developed malignancies than female (33/1802) (ratio  = 1.44) [6]. However, Kummer *et al*. recently suggested that both male and female CKD patients have the same poor survival once dialysis therapy has been initiated [26]. They proposed that the increased progression of renal outcomes for females may be due to genetic factors and gender hormones. Advanced analysis of the interaction of gender effect and dialysis modality on ESRD-related malignancies is rarely proposed in literature. Therefore, we want to explore the effect of sex difference and the interaction of sex and dialysis modality on the incidence of the risk of ESRD-related malignancies. In the present analysis, a higher risk of overall cancer morbidity was seen among female ESRD patients than among males. The results were in accordance with previous findings obtained by other investigators [22], [24]. According to former literatures, a higher percentage of females than males preferred alternative therapies, including Chinese herbs containing aristolochic acid. In a Taiwan cohort with 6,548 Chinese herbalists, female herbalists were at higher risk for urologic cancers than males (standardized mortality ratio (SMR) = 3.10; 95% CI = 1.41–5.87) [27]. Another large prospective cohort study in Taiwan demonstrated a significantly increased risk of CKD among regular users of Chinese herbal medicines [28]. This hypothesis was in accordance with that of a Belgian cohort [29]. In a female rabbit model, chronic exposure to aristolochic acid, given as a single drug, casually induced renal hypocellular interstitial fibrosis, and even the development of urinary tract tumors [30]. Another Taiwanese study indicated that young women taking Mu-Tong and Fangchi (aristolochic acid–related herbal products) and over the counter drugs were more likely to develop transitional cell carcinoma, chronic tubulointerstitial nephritis [31], and renal failure, especially those less than 50 years old [32]. Further studies to verify the difference in cancer risk between males and female are needed. We also demonstrated that female patients undergoing RT or PD had higher risks of RCC and UC. The underlying mechanisms require further elucidation.

Another issue that warrants discussion is the possible gender difference in the competing risk mortality in the present study. The progression and mortality of ESRD may compete before the incidence of ESRD-related malignancies. In general, males have a higher mortality from cardiovascular disease than females [33]. However, according to the ANZDATA 2010 Annual Report, cancer is surpassing cardiovascular diseases as the leading cause of post-transplantation death [34]. Among our 17,850 study subjects, the average follow-up durations stratified by gender are 3.50 and 3.25 years in females and males with developed cancer, respectively, as well as 4.39 and 4.08 years in females and males without developed cancer (data not shown). The average follow-up from the date of recruitment through surrender stratified by gender are similar, i.e., 4.43 and 4.11 years in females and males, respectively (data not shown). The similar follow-up periods of males and females may imply the low effect of competing risk mortality. Although the average follow-up period of recruitment to surrender may not represent the actual mortality rate, we believe that it is the approaching index in the present analysis. All ESRD patients who received any kind of dialysis modality should be recorded in the registry of catastrophic illness patients. Other than death, no other important reason to surrender is observed. However, further investigative studies are needed to confirm the links among the sex differences, mortality of competing risk, and incidences of ESRD-related malignancies.

A retrospective cohort study of the present study was used to evaluate cancer morbidity using population-based registries to avoid ascertainment bias during long-term follow-up. Morbidity rather than mortality was better for exploring the correlation between ESRD and all-site cancer because the mortality rate might be underestimated considering the risk can be improved by early screening and by increasing medical intervention. We also adopted frequency matching to balance the distributions of age, gender, and index date of ESRD diagnosis in the ESRD and non-ESRD groups to increase the comparability. The limitations of the current study are as follows: First, the limited sample size of people receiving PD or RT treatment resulted in a confidence interval too extensive to acquire meaningful conclusions or to execute stratified analyses. In addition, people receiving RT in foreign country could not recruit in the present analysis. Second, the related laboratory measurements or personal lifestyle risk factors were not included in the imbursement database. Therefore, the effects of potential covariates on the correlation between dialysis modality and cancer risk could not be evaluated.

In summary, ESRD is associated with an increased morbidity of all-site cancer regardless of different RRT modality, especially for female patients. Multicenter recruitment and measurements of laboratory data might be of benefit to have a sufficiently large sample size for stratification analysis and to explore further the possible mechanism of sex differences for ESRD-related cancer morbidity.
